# The Effect of Acute Pre-Workout Supplement Ingestion on Basketball-Specific Performance of Well-Trained Athletes

**DOI:** 10.3390/nu15102304

**Published:** 2023-05-14

**Authors:** Athanasios Douligeris, Spyridon Methenitis, Antonia Lazou, George Panayiotou, Konstantinos Feidantsis, Gavriela Voulgaridou, Yannis Manios, Athanasios Z. Jamurtas, Constantinos Giaginis, Sousana K. Papadopoulou

**Affiliations:** 1 Department of Nutrition Sciences and Dietetics, Faculty of Health Sciences, International Hellenic University, 57400 Thessaloniki, Greece; smetheni@phed.uoa.gr (S.M.); kfeidant@bio.auth.gr (K.F.); gabivoulg@gmail.com (G.V.); 2 Sports Performance Laboratory, School of Physical Education & Sports Science, National and Kapodistrian University of Athens, 15772 Athens, Greece; 3 Theseus, Physical Medicine and Rehabilitation Center, 17671 Athens, Greece; 4 Nutrition and Dietetics, School of Life and Medical Sciences, University of Hertfordshire, Hatfield AL109AB, UK; lazouantonia@gmail.com; 5 Laboratory of Exercise, Health and Human Performance, Applied Sport Science Postgraduate Program, Department of Life Sciences, School of Sciences, European University Cyprus, 1516 Nicosia, Cyprus; g.panayiotou@euc.ac.cy; 6 Department of Nutrition and Dietetics, School of Health Sciences and Education, Harokopio University, 17671 Athens, Greece; manios@hua.gr; 7 Institute of Agri-food and Life Sciences, Hellenic Mediterranean University Research Centre, 71410 Heraklion, Greece; 8 Department of Physical Education & Sport Science, University of Thessaly, 42100 Trikala, Greece; ajamurt@uth.gr; 9 Department of Food Science and Nutrition, School of Environment, University of the Aegean, 81400 Myrina, Greece; cgiaginis@aegean.gr

**Keywords:** caffeine, creatine, nitric oxide, β-alanine, L-citrulline, branched chained amino acids jumping, sprint, agility, anaerobic performance, aerobic performance

## Abstract

A pre-workout supplement’s (PWS; 200 mg caffeine, 3.3 g creatine monohydrate, 3.2 g β-alanine, 6 g citrulline malate and 5 g branched chained amino acid (BCAA) per dose) acute effects on the alactic (jumping, sprinting, agility), lactic (Running-Based Anaerobic Sprint Test, RAST) and aerobic performance (Yo-Yo Intermittent Recovery Test Level 1, Yo-Yo IRL1 VO_2max_) of well-trained basketball players was investigated in this double-blind placebo-controlled study. Thirty players (age 18–31 years, height 166–195 cm, weight 70.2–116.7 kg, body fat 10.6–26.4%) were allocated to pre-workout (PWS, *n* = 15) or placebo (PL, *n* = 15) groups. Half of the participants in each group performed the evaluations without PWS or PL, while the rest consumed PWS or PL 30 min before the assessments (1st trial) and vice versa (2nd trial). Significant improvements in counter-movement jump (CMJ) (PWS: 4.3 ± 2.1%; PL: 1.2 ± 1.0%), agility (PWS: −2.9 ± 1.8%; PL: 1.8 ± 1.7%), RAST average (PWS: 18.3 ± 9.1%; PL: −2.2 ± 2.0%), minimum power (PWS: 13.7 ± 8.9%; PL: −7.5 ± 5.9%), and fatigue index (PWS: −25.0 ± 0.9%; PL: −4.6 ± 0.6%) were observed in the PWS group vs. the PL group (*p* < 0.05). No differences were found regarding sprinting, aerobic performance, and blood lactate concentrations. Thus, although players’ alactic and lactic anaerobic performance could be improved, peak power, sprinting and aerobic performance are not.

## 1. Introduction

Numerous studies have reported athletes’ readiness and performance improvement after the consumption of caffeine (3–6 mg/kg body mass) [1], creatine (3–5 g) [2], β-alanine (4–6 g) [3], nitric oxide agents (e.g., citrulline malate (6–8 g)) [4] or amino acids (usually branched chained amino acids (BCAAs) (~5.6–20.0 g)) [5,6], before or during training or competition [7,8,9,10,11]. Caffeine (a nervous system stimulant) blocks adenosine receptors and is involved in motor unit firing rates, pain attenuation, and fatigue delay [1]. Creatine supplementation increases the respective muscle creatine stores, enhances the phosphocreatine resynthesis rate, and sustains adenosine triphosphate (ATP) availability during maximal anaerobic sprint exercise [2]. Furthermore, creatine supplementation may improve exercise capacity, delay fatigue and promote greater training adaptations during short-term high-intensity training [2]. Β-alanine supplementation increases the intramuscular carnosine concentration and acts as an intracellular pH buffer to attenuate exercise-induced acidosis [3]. L-citrulline supplementation raises plasma citrulline and arginine concentrations, promoting nitric oxide (NO) systemic availability [12], and may aid in ammonia detoxification via the urea cycle, decrease lactate production, and improve aerobic utilization of pyruvate, thus enhancing muscular function and decreasing fatigue [4]. NO manages skeletal muscle and blood vessel functions (e.g., enhancing vasodilation and calcium handling in the sarcoplasmic reticulum) that may improve exercise performance [12]. L-citrulline is commonly supplemented in the form of citrulline malate since it appears that citrulline and malate may work synergistically to enhance skeletal muscle tissue perfusion and the efficiency of ATP production to optimize exercise performance [4]. Finally, parallel to the above, BCAA intake attenuates muscle fatigue during prolonged exercise and improves performance by inhibiting free tryptophan transport to the brain and reducing serotonin concentration levels [6,11]. However, the potential benefits of BCAA for muscular performance are unclear and warrant further research to assess the impact of BCAA on athletic performance [13,14].

Based on findings related to the independent effects of each of the above supplementation on athletes’ performance, currently, athletes’ consumption of pre-workout supplements (PWSs) has increased. PWSs are formulas that usually contain different combinations of the above ingredients. Theoretically, the above ingredients may work synergistically when consumed together, improving several aspects of athletes’ performance [15]. PWS consumption in recreationally or physically trained males leads to many performance-enhancing benefits, including improvements in mean power output during single and repeated sprints [15,16], agility, reaction times, lower body muscular endurance and reduced fatigue [17,18]. PWS ingestion also improves anaerobic performance [19] and prolongs time to exhaustion during high-intensity intermittent exercise [20]. In addition, PWSs seem to increase the capacity for greater training volumes, muscular endurance, and power performance throughout a training session [15,16]. However, PWSs’ effectiveness is not constant, as they do not alter anaerobic power [21,22,23], jumping performance [23,24] or blood lactate concentrations after a training session, at least in recreationally trained males and strength-power athletes. Moreover, the effects of long-term PWS supplementation, where some of these agents were combined (e.g., β-alanine, creatine, citrulline malate, etc.) to assess endurance-trained runners or elite cyclists’ performance, are mixed and less clear [25,26]. This inconsistency may rely on the fact that each PWS varies in ingredients’ type and dosage. Furthermore, caffeine, one of the main ingredients in PWSs, may work differently depending on users’ dose and habitual consumption [27]. Additionally, many ingredients in the most commonly used commercial PWSs are either below the effective dosage range or labelled as proprietary blends with unspecified proportions of each ingredient [28]. For instance, it has been reported that the average amounts of beta-alanine (2.0 ± 0.8 g) and citrulline (4.0 ± 2.5 g) per serving size in PWSs are significantly below the recommended effective dose range (eff. range: β-alanine 4–6 g, citrulline 6–8 g) [3,4], whereas the average caffeine dose is closer to the lower limit of the effective dose (effective range: caffeine 3–6 mg/kg of body weight) [1].

Even though the popularity of PWS use has increased among trained/professional athletes, most of the data in this area are derived from recreationally and not from well-trained athletes of a competitive level (especially in team sports) [29]. Basketball is classified as an intermittent type–mixed aerobic and anaerobic activities sport [30]. In particular, it includes short bursts of high-intensity activities, such as sprinting, rapid changes of direction, acceleration, deceleration, shuffling, backwards running and jumping, followed by short bursts of low-intensity activity, such as walking, jogging, and short periods of recovery [31]; therefore, it engages high requirements of anaerobic/aerobic capacities [32,33,34]. To the best of our knowledge, only one study evaluated the acute effects of PWSs on aerobic and anaerobic performance in college basketball players [35], according to which the consumption of a PWS containing caffeine (210 mg), creatine monohydrate (1.5 g), arginine (3 g), citrulline (1 g), β-alanine (2.8 g), BCAA (3 g), taurine (800 mg) and tyrosine (200 mg), 60 min prior to the performance evaluations, exhibited a positive effect on jumping and anaerobic performance, but exhibited no effect on the fatigue index or aerobic performance [35].

To the best of our knowledge, only scarce data exist regarding the evaluation of the effectiveness of PWSs on basketball players’ performance, and the acute effect of PWSs on other important physical performance parameters of basketball players, such as agility and speed [31], has never been investigated until now. Therefore, the present study aimed to examine the acute effects of a PWS, containing 200 mg caffeine, 3.3 g creatine monohydrate, 3.2 g β-alanine, 6 g citrulline malate and 5 g BCAA per dose, on jumping, sprinting, agility, and aerobic and anaerobic performance in well-trained basketball players. The PWS dosage consisted of the most common mix of ingredients reported in the literature, with the intention to remain within the effective dosage range of each ingredient [6,7,8,9,10,11] and under 100 kcal/serving to avoid any gastrointestinal discomfort during exercise. It was hypothesized that sprinting, jumping, agility, and anaerobic performance would increase acutely after this PWS blend’s consumption.

## 2. Materials and Methods

### 2.1. Study Design

The present study was a double-blind, placebo-controlled, counterbalanced one consisting of PWS and placebo (PL) treatments. Four field-based anaerobic basketball tests: jumping, sprinting, agility, Running-Based Anaerobic Sprint Test (RAST); and an aerobic test: Yo-Yo Intermittent Recovery Test Level 1 (Yo-Yo IRL1 VO_2max_), were performed [31]. Participants were recruited from local basketball teams competing in a Greek national first-division championship. The inclusion criteria were: (1) male participants, (2) no restraining orthopedic/neuromuscular problems, (3) no caffeine hypersensitivity (as assessed by the health history questionnaire (HHQ)), (4) no consumption of more than three servings of coffee per day, (5) no use of any PWS or steroids prior to the study, (6) no use of creatine, β-alanine, citrulline malate or BCAA-protein supplementation at least six months before the initiation of the present study, (7) absence of drugs, abuse or medications, which are known to affect the sprinting, jumping, agility, anaerobic and endurance performance, and (8) consent to follow a regular dietary plan for basketball players. Volunteers fulfilling the inclusion criteria visited our laboratory for their medical examination screening and stratification. At the same time, after receiving a detailed verbal explanation and written instructions from our team’s registered nutritionists, they were asked to complete a 3-day nutritional recall questionnaire before the start of the initial measurements to determine their nutritional behavior–energy intake. Participants were instructed to follow the exact same diet 3 days before the onset of the subsequent measurements, one week apart. During the second visit, all participants were familiarized with the evaluations of their alactic (jumping, sprinting, agility), lactic (RAST) and aerobic (Yo-Yo IRL1 VO_2max_) performance. One week later, participants visited the basketball court between 9:00–10:00 am and were assigned into two groups (PWS or PL; *n* = 15 per group). During this visit, half of the participants in each group performed the evaluations without PWS or PL. At the same time, the rest consumed PWS or PL 30 min before the assessments, according to their group. The evaluations were performed according to National Strength and Conditioning Association (NSCA) standards [31]. They were: (1) body composition and resting heart rate blood pressure, (2) counter-movement jump (CMJ), (3) 20 m sprint, (4) agility T-Test, (5) Running-Based Anaerobic Sprint Test (RAST) and (6) Yo-Yo Intermittent Recovery Test Level 1 (Yo-Yo IRL1 VO_2max_). Between the evaluation of RAST and Yo-Yo IRL1 VO_2max_, a 30 min rest interval was applied, while the rest period between all other assessments was 10 min. One week later, all participants proceeded to the same basketball court at the same hours and completed all the above evaluations in the same sequence; however, on a reversal supplementation schedule, volunteers who had previously consumed PWS or PL did not consume anything, whereas those who had previously received nothing now received PWS or PL 30 min before the evaluations, depending on their group. On both occasions, before the initiation of performance evaluations, all participants underwent a 15 min standardized warm-up consisting of low-intensity running and dynamic lower-body stretching. The present study took place during the in-season period, and all participants were instructed to avoid alcohol or caffeine consumption for at least 24 h before each testing session. Intra-class correlation coefficients (ICC) of each evaluation were determined in a pilot study, prior to the initiation of the study, in an aged and performance matched group of 10 basketball players, which did not participate in data collection.

### 2.2. Participants

Thirty active basketball players volunteered to participate in this study. Daily activity levels indicated that participants exercised frequently and regularly (training sessions per week, 5.7 ± 0.6; training session duration 1.5 ± 0.1 h; resistance training sessions per week 2.5 ± 1.2; and resistance training duration 1.0 ± 0.4 h). All individuals completed health history and exercise questionnaires before enrolling in the study and signed a written informed consent after all methods, risks, and benefits were thoroughly explained. A research member not involved in data collection used the block method [36] to allocate participants into two groups to ensure a double-blind approach and avoid reporting bias [37]. The two groups were: (A) PWS and (B) PL (Table 1). All procedures relative to this study were in accordance with the Helsinki Declaration and approved by the International Hellenic University ethics committee for the protection of human subjects (ref. number 07/09.06.2022).

### 2.3. Performance Tests and Measurements

#### 2.3.1. Body Mass, Height, Composition, Resting Heart Rate and Blood Pressure

Height was measured using a stadiometer (Leicester portable height measure Tanita HR 001, Tokyo, Japan). Body mass, body fat percentage, body fat mass and fat-free mass were measured using a calibrated digital scale to the nearest 0.1 cm and 0.1 kg and analyzed via Bioelectrical impedance analysis (Tanita BF-522W, Tokyo, Japan), according to manufacturer instructions (the intraclass correlation coefficient (ICC) ranged between 0.93 and 0.98). An upper body blood pressure monitor was used to measure blood pressure and heart rate (Omron M2 Basic, Kyoto, Japan; ICC: 0.91–0.95).

#### 2.3.2. Jumping Performance

Counter-movement jump (CMJ) performance was evaluated using a professional contact mat, which contains sensors that assess the strength exerted by the feet (CM 60 × 43, ALGE-TIMING, Lustenau, Austria), according to previously described protocols [38,39,40,41]. After a standard warm-up described above, athletes performed 3 CMJs with submaximal effort. After 3 min of rest, players performed three maximal attempts (with hands placed on hips), with 3 min rest between them. The best performance was retained for statistics (ICCs: 0.91 and 0.93, respectively).

#### 2.3.3. Sprint Performance

Sprint running performance, i.e., the shortest time needed to cover the distance of 20 m, was measured on a basketball court in accordance with previous reports [38,39,40,41]. The time was recorded using photocell gates (Photocell PR1aW, ALGE-TIMING, Lustenau, Austria, accuracy of 0.01 s) 0.4 m above the ground, set on the start (0 m) and finish (20 m) lines, with reflectors at 1 m. Participants initially performed three submaximal sprints, with three minutes of rest between them, and after 5 min, they performed three maximal sprints (standing start position), separated by 3 min intervals [38,39,40,41]. The best performance was retained for statistics (ICC = 0.91).

#### 2.3.4. Agility T-Test (ATT)

Agility is a skill that should be considered and developed in basketball players [42] since it has been recognized as a physiological prerequisite for successful performance in basketball [31]. The ATT consists of 4 multidirectional, basketball-specific movements: sprinting, lateral shuffling, and backwards running. It examines how quickly athletes can change direction and evaluates the agility of a basketball player [33,43]. For the purpose of this study, a standard protocol outlined for ATT was implemented [44,45]. The participants completed three t-test routines on a wooden basketball court, with five-minute rests apart. The test time was recorded using photocells gates (Photocell PR1aW, ALGE-TIMING, Lustenau, Austria, accuracy of 0.01 s), and the best performance was further used for statistics (ICC: 0.98).

#### 2.3.5. Running-Based Anaerobic Sprint Test (RAST)

RAST seems to be a reliable test for measuring basketball players’ anaerobic capacity [46], with a test reliability coefficient of 0.88 [47]. The basketball-specific RAST included six maximal 35 m round-trip runs separated by two 17.5 m shuttle runs with 10 s rest intervals [48]. Photocell gates (Photocell PR1aW, ALGE-TIMING, Lustenau, Austria, precision of 0.01 s) were placed 0.4 m above the ground to determine the timing of each sprint. Following the completion of the test, the following variables were calculated: Power = Weight (kg) × Distance (m^2^) ÷ Time (s^3^). Maximum power = the highest value of six sprints, Minimum power = the lowest value of six sprints, Average power = sum of all six values ÷ 6, Fatigue Index (FI) = (peak power − minimum power/peak power) × 100. The ICC for these evaluations ranged from 0.89 to 0.94.

#### 2.3.6. Yo-Yo Intermittent Recovery Test Level 1 and Blood Lactate Concentrations

Increased aerobic capacity influences basketball performance and recovery [33], allowing the athlete to perform several high-intensity activities during the game [49]. The Yo-Yo intermittent recovery test level 1 (Yo-Yo IRL1 VO_2max_) is an excellent tool to evaluate aerobic capacity [33] in the field [50]. The Yo-Yo IRL1 VO_2max_ test consisted of multiple 40 m stages of progressively increased speed, controlled by audio signals, at which, in each stage athletes must run 20 m from the starting line, turn and run again for 20 m until the starting line. Between stages, athletes have a 10 s period of active recovery (2 × 5 m of jogging). The test began at 10 km/h and gradually increased by 0.5 km/h with each round, and ended when the participant voluntarily quit or failed to complete the shuttle run in time on two consecutive occasions due to fatigue [51]. The total distance achieved during the Yo-Yo IRL1 VO_2max_ was measured to calculate maximum oxygen consumption (VO_2max_). VO_2max_ was assessed from the following equation: VO_2max_ (mL/min/kg) = IR1 distance (m) × 0.0084 + 36.4 [51]. The ICC of this test ranges from 0.78 to 0.98. Blood lactate concentration (BL) (mmol/L) was measured using a portable blood lactate analyzer (Lactate Pro 2, LT-1730; Arkray, Kyoto, Japan) from a finger prick blood sample (0.3 mL) three minutes after the end of the Yo-Yo IRL1 VO_2max_ test [52].

#### 2.3.7. Supplementation

The PWS developed for this study (BIONATIV SRL, Bucharest, Romania) consisted of 200 mg caffeine (2.4 ± 0.4 mg/kg of body weight), 3.3 g creatine monohydrate, 3.2 g β-alanine, 6 g citrulline malate and 5 g BCAA per dose to remain close to the recommended effective dose range (caffeine: 3–6 mg/kg body mass, creatine: 3–5 g/day, β-alanine: 4–6 g/day, citrulline malate: 6–8 g/day, BCAA: 5–10 g/d), and under 100 kcal/serving to avoid any gastrointestinal issues during exercise [1,3,4,53,54]. However, the dose of caffeine, taking into account the study group, was significantly lower (2.4 ± 0.4 mg/kg of body weight), and in some participants did not exceed 2 mg/kg of body weight. The PWS group consumed one scoop (20 g, ~73 Kcals, tropical fruit flavor) of powder diluted in 300 mL water. The PL group also consumed one scoop (20 g, ~74 Kcals, 97% flavored maltodextrin, similar in color, flavor, taste and energy as PWS) of powder diluted in 300 mL of water. To ensure a double-blind approach, a study member that was not involved in any data collection prepared both supplement and placebo drinks for all participants. Participants were allowed to drink cold water ad libitum 30 min before the initiation of the performance evaluations.

### 2.4. Statistical Analyses

A post hoc power analysis (G*Power ver 3.1; FrankFaul, Universitat Kiel, Kiel, Germany) was performed according to the study design, the number of participants and the lower partial eta squared (η^2^) of the significant contrasts, which reveal an actual power of 0.903 for the results of the present study. The Shapiro–Wilk test was employed to test the normality of data. All data are presented as means and standard deviation (mean ± SD). Two-way repeated measures analysis of variance (and, when appropriate, Bonferroni Post hoc test) was used to investigate differences between the groups. η^2^ was evaluated and used as an indicator of effect size, and it could be classified as small (0.01 to 0.059), moderate (0.06 to 0.137) and large (≥0.138). The magnitude of effect size (ES) for the results within each group was determined by Cohen’s d [55], which in trained athletes below 0.20 is considered trivial, between 0.21–0.50 as small, between 0.51–1.00 as moderate and above 1.00 as large [56]. Paired samples T-Test was used to compare the percentage changes (% of pre- to post-changes) in each variable between the groups. Statistical analyses were performed with SPSS Statistics Ver. 20 (IBM Corporation, New York, NY, USA), while the significance level was set at *p* ≤ 0.05.

## 3. Results

**Figure 1:**
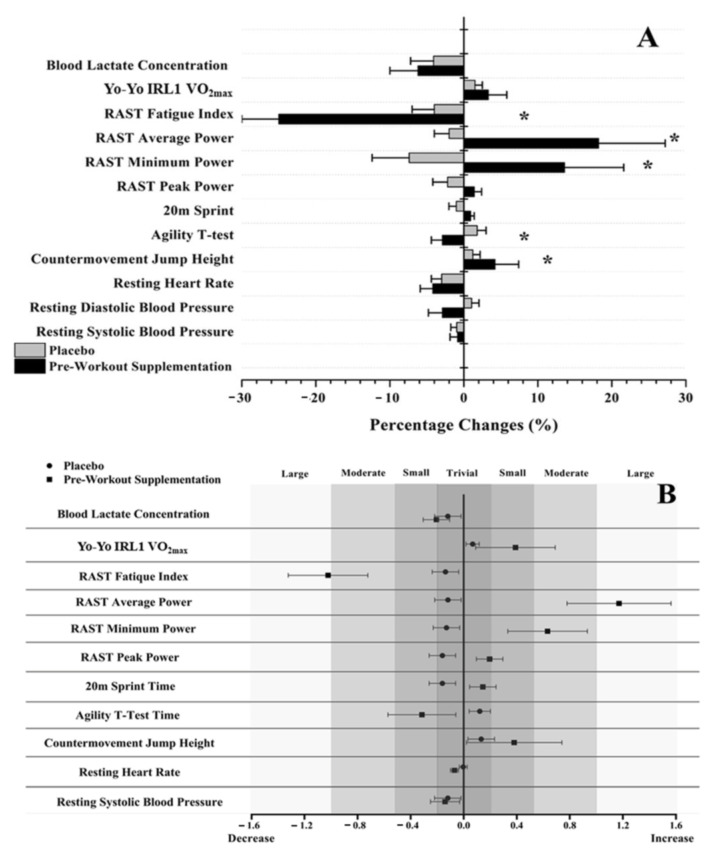
(**A**) Pre- to post-intervention percentage changes (%) in body composition, resting heart rate, blood pressure and performance. (**B**) Cohen’s d values (effect size) of the same parameters for each group, with error bars, represent 95% confidence limits and their categorization. Asterisk (*) indicates *p* < 0.05 between groups.

No significant differences between the groups were found for the baseline values (*p* > 0.425; η^2^ < 0.004). No significant differences between and within (days of evaluation) the groups were also found regarding their nutrition (*p* > 0.625). Significant differences were observed in CMJ (d = 0.31; *p* = 0.019) and agility T-test (d = −0.32; *p* = 0.018) only in the PWS group. Furthermore, significant changes were found in RAST average power (d = 1.12; *p* = 0.039), minimum power (d = 0.63; *p* = 0.019) and fatigue index (d = −1.32; *p* > 0.001) in the PWS group only. No significant differences were observed in both groups’ body mass and composition, 20 m sprint test, RAST peak power, Yo-Yo IRL1 VO_2max_, blood lactate concentrations, and resting systolic, diastolic blood pressure and heart rate (*p* > 0.005; Table 2 and Figure 1). No significant differences between trials were found in the PL group (*p* > 0.356).

## 4. Discussion

The main findings of the present study were that the acute PWS consumption consisting of 200 mg caffeine, 3.3 g creatine monohydrate, 3.2 g β-alanine, 6 g citrulline malate and 5 g BCAA per dose exhibited an acute positive effect on the counter-movement jump (CMJ), agility performance, RAST average power, minimum power and fatigue index in well-trained basketball players. In contrast, this PWS did not affect sprinting, RAST peak power, aerobic performance, blood lactate concentration, resting blood pressure and heart rate.

### 4.1. Resting Blood Pressure and Heart Rate

According to the results of the present study, no significant changes were observed in resting blood pressure or heart rate between participants who consumed the PWS before exercise and those who consumed a placebo. Caffeine consumption may abruptly elevate blood pressure and heart rate, with peak levels occurring thirty minutes after oral administration and lasting up to two hours [57]. We can assume that the reason for these observations may rely on the acute nature of our intervention [57].

### 4.2. Jumping Performance

The results of the present study indicate that acute PWS consumption could induce significant but minor improvements in the jumping performance of basketball players. The improvement in jumping performance observed in our study could be attributed to caffeine ingestion rather than creatine, β-alanine, citrulline malate, or BCAAs, as these regiments seem to improve athletes’ power production only after long-term supplement administration [26,58]. Acute caffeine consumption improves jumping performance by enhancing athletes’ ability to produce greater peak force and rate of force development during a counter-movement jump [59]. Indeed, Lazić et al. [7], in a recent meta-analysis, reported that the consumption of caffeine seems to be effective in the acute improvement of vertical jump performance in basketball players. On the other hand, it appears that acute consumption of creatine, β-alanine, citrulline malate or BCAAs has little to no influence on peak force and rate of force development in the absence of chronic loading or acute consumption associated with jumping height [23]. Our research findings align with Çetin et al. [35], who reported significant increases in CMJ performance after acute PWS ingestion in college basketball players. In contrast, acute PWS consumption does not seem to significantly affect college football players or recreational handball male players’ jumping performance [15,24]. This discrepancy could be explained by the fact that basketball players perform more vertical jumps during training and matches than football or handball players [30].

### 4.3. Sprinting Performance

Acute PWS consumption does not seem to improve sprinting performance in the basketball players investigated herein. However, the acute effect of a PWS containing many ergogenic ingredients (i.e., caffeine, creatine, β-alanine, citrulline malate, BCAAs) on basketball players’ sprinting performance has never been investigated. Caffeine can act as an adenosine antagonist [1], which reduces adenosine’s capacity to downregulate central nervous system arousal [60], and increase muscle fibers’ firing rates [61]. In contrast to the results of the present study, it has been reported that caffeine, when ingested prior to training sessions, could lead to significant but minor improvements in 10 m and 20 m sprinting performance in basketball players [7]. The above differences could be attributed to the fact that the effect of caffeine on athletes’ performance is not constant. For example, significant inter-individual differences between basketball players in their response to caffeine administration have been reported since many do not demonstrate any improvement in their sprinting performance after acute caffeine consumption [62]. Furthermore, several reports indicate that the impact of acute consumption of caffeine has either low or trivial improvement in athletes’ sprinting performance in team sports [63]. This disparity is most likely due to the differences in movement patterns found in each sport activity. However, the underlying mechanisms provoking these differences between professional athletes should be investigated in future studies. Finally, it appears that in the absence of long-term supplementation, the consumption of creatine and β-alanine and acute ingestion of citrulline malate and BCAAs exhibits no influence on sprinting performance [2,17,64].

### 4.4. Agility Performance

The agility performance of basketball players seems to exhibit a significant but small acute improvement following the consumption of such PWSs. The acute effect of a PWS (containing caffeine, β-alanine, creatine, citrulline malate and BCAAs) on agility performance in basketball players has never been examined. Caffeine [1] appears to be the primary reason for such an acute improvement since the other components employed in the present study’s PWS (for instance, creatine and β-alanine) seem to require a more extended administration period for significant effects to be observed [65,66]. Indeed, according to a recent meta-analysis, acute caffeine supplementation 60 min before exercise at a dosage of 3–6 mg per body mass induces significant, acute improvements in athletes’ agility performance [67]. In contrast, even though creatine supplementation tends to improve athletes’ agility performance, its effect is not acute and requires at least one week of systematic administration for significant improvements in agility performance to be observed [68]. However, consumption of a PWS containing L-citrulline, β-alanine, taurine, L-arginine, L-tyrosine and anhydrous caffeine (~2.7 mg/kg of BW) could induce greater improvements in recreational handball male players’ agility performance compared to only caffeine supplementation consumption [24]. According to Kaczka et al. [24], the reported improvements may be the outcome of the synergistic actions of all ingredients that the employed PWS contained instead of caffeine alone (~2.7 mg/kg of BW) [24]. The latter may also be valid for the observations in the present study since the dose of caffeine consumed by PWS group in our study, was significantly lower (2.4 ± 0.4 mg/kg of body weight). Thus, considering the above, it seems that the agility performance of well-trained basketball players may be acutely enhanced after the PWS consumption. However, such an effect was not observed in the sprinting test, which used the exact alactic anaerobic mechanism. This discrepancy may be partly explained by the fact that the mean time required to complete the agility test was ~10.5 s instead of ~3.1 s in sprinting test, and the longer duration of the agility test was the main factor for such an improvement.

### 4.5. RAST Performance

The consumption of such a PWS seems to significantly induce moderate to considerable acute improvements in average power, minimum power, and fatigue index, but no improvements in peak power during RAST. Probably the improvement observed in power and fatigue indexes is attributed to caffeine, citrulline malate and BCAAs consumption included in this PWS and not to creatine and β-alanine. Caffeine seems to be one of PWS’s main ingredients that could improve repeated sprint velocity, number of sprints [63] and muscle power [69]. The mechanisms behind these improvements seem to be the adenosine receptors antagonism, the enhanced release of certain neurotransmitters, the increase in catecholamine levels [67], and finally, the increase in neuromuscular function [70]. Caffeine consumption has also been linked to a reduction in the magnitude of perceived effort during high-intensity cycling [71], which could partially explain the improvement in the fatigue index observed in the present study. Citrulline malate is another compound in this PWS that could positively affect power, anaerobic performance and resistance to fatigue. In a recent meta-analysis, it has been reported that the acute ingestion of citrulline malate, in a range of 6–8 g one hour before exercise, could increase athletes’ capacity for greater training volume and lower neuromuscular-metabolic fatigue during high-intensity activities [12]. The latter could be attributed to enhanced oxygen uptake, muscles’ aerobic metabolism, the delivery of nutrients to the muscles [72], muscles’ buffer capacity [73], and post-exercise perceived effort and muscle soreness [74]. BCAAs, probably through the enhancement of muscle metabolism and protection against muscle damage, also positively affect anaerobic performance [11]. Moreover, the synergistic effect of BCAAs, arginine and citrulline attenuates exercise-induced central fatigue in top-level athletes, highlighting the importance of those substances when consumed together [75]. In contrast, it seems that the acute consumption of creatine and β-alanine does not contribute to the acute improvement of anaerobic performance since significant gains in anaerobic and power performance and delay of neuromuscular fatigue onset have been reported only after chronic administrations [3,9]. It is, therefore, unlikely that the improvements observed in the present study regarding anaerobic performance and fatigue index rely on creatine and β-alanine consumption. The present study’s results align with Panayi et al. [18], who also reported that acute PWS consumption containing caffeine, citrulline malate, creatine, β-alanine, and amino acids before exercise might reduce muscular fatigue and enhance anaerobic power output during repeated high-intensity cycling in recreationally trained individuals. In contrast, Çetin et al. [35], reported significant improvements after PWS ingestion in relative peak and average power, but not in fatigue index in basketball players. This discrepancy may be attributed to a higher level of citrulline malate and BCAAs in the PWS used in the present study compared to the one of Çetin et al. [35]. Citrulline malate in a single acute dose of 6 g, similar to that used in our PWS, seems to benefit fatigue and muscle endurance during high-intensity exercise [12]. Still, lower doses may be ineffectual in eliciting such an ergogenic response. Furthermore, the higher BCAA levels in this PWS (5 g vs. 3 g in Çetin et al. [35]) appear to be more beneficial in the prevention of muscular fatigue [76].

### 4.6. Aerobic Performance

No significant changes in aerobic performance (Yo-Yo IRL1 VO_2max_) have been found, following PWS consumption, exhibiting that a single dose of caffeine, creatine, β-alanine, citrulline malate, and/or BCAAs is inadequate to promote significant improvements in Yo-Yo IRL1 VO_2max_. Acute caffeine consumption (3–6 mg/kg of body weight) [77], or oral ingestion of 20 g of BCAAs one hour before the exercise [6], exhibits a small but positive effect on endurance performance. However, the caffeine amount in the current supplement in the PWS group was 2.4 ± 0.4 mg/kg of body weight, and the BCAAs content was 5 g, which may be too low to induce any noticeable influence on aerobic performance. Furthermore, acute citrulline malate [78], creatine, and β-alanine consumption [26] are ineffective in promoting aerobic performance without chronic loading. Our study’s findings align with Çetin et al. [35], who found that PWS consumption exhibited no acute effect on VO_2max_ in basketball players during the same test. Furthermore, Lutsch et al. [79] reported no significant difference between the effect of a PWS or placebo supplementation on running performance during a 5 km race in aerobically trained participants. Our results also indicated no significant effect on blood lactate concentration three minutes after exercise. These findings are in accordance with the results of Jagim et al. [15], who found that PWS consumption had no significant effect on blood lactate concentration after exercise.

### 4.7. Side Effects

No significant side effects (e.g., gastrointestinal issues) were observed or reported from the athletes during the study. The only side effect that it has been reported in few athletes of PWS group (2 participants) was a tingling sensation in the neck and the backside of the hands. The tingling sensation is a common side effect of β-alanine consumption in high doses, lasting approximately 60 min. Still, to date, no scientific evidence supports that this tingling sensation is dangerous in any way [3,80].

### 4.8. Study’s Limitations

The first limitation of the present study was that we did not investigate the acute effect of each ingredient separately on basketball performance. The second limitation was that the physiological background (blood samples, metabolic pathways, etc.), which could explain the results of the present study, was not evaluated. The third limitation was that the dose of caffeine in the study group, was significantly lower (2.4 ± 0.4 mg/kg of body weight) than the recommended dose of caffeine (3–6 mg/kg of bodyweight) and, in some participants, did not exceed 2 mg/kg of body weight. Furthermore, the dose of BCAA was also low. Finally, the present study report only the acute effect of this PWS supplementation. Given that most supplementation needs a more extended period of administration to act, future studies should investigate the longitudinal effect of such PWSs on basketball players’ performance.

## 5. Conclusions

Considering the limitations of the present study, the results of the present study suggest that consumption of a PWS containing 200 mg caffeine, 3.3 g creatine monohydrate, 3.2 g β-alanine, 6 g citrulline malate and 5 g BCAA per dose 30 min prior to training or match could acutely improve jumping and agility alactic performance, as well as lactic anaerobic performance and fatigue index in well-trained male basketball players, without any changes on their resting blood pressure and heart rate. However, this supplement seems insufficient for acute peak power, sprinting and aerobic performance improvements. Thus, using such PWS appears promising and should be encouraging, prior to a training session or match, when an acute increase in players jumping, agility and anaerobic performance is necessary.

## Figures and Tables

**Table 1:** Anthropometric data.

	Pre-Workout Supplement (*n* = 15)	Placebo (*n* = 15)
Age (yrs)	23.7 ± 5.7	26.7 ± 7.2
Height (cm)	183.8 ± 7.6	187.4 ± 5.5
Body Mass (kg)	83.7 ± 14.2	87.9 ± 8.5
Fat-Free Mass (kg)	14.1 ± 6.1	15.6 ± 4.8
Fat Mass (kg)	69.5 ± 8.8	72.3 ± 6.9

Data presented as mean  ±  SD.

**Table 2:** Resting heart rate, blood pressure and performance indices before and after pre-workout or placebo supplementation.

	Pre-Workout Supplement (*n* = 15)		Placebo (*n* = 15)		η^2^/*p*		
	Pre	Post	Pre	Post	Time	Group	Time × Group Interaction
Body Composition, Resting Heart Rate and Blood Pressure							
Resting Systolic Blood Pressure (mmHg)	123.3 ± 7.9	122.3 ± 7.1	124.0 ± 9.4	122.8 ± 7.2	0.092/0.089	0.003/0.763	0.007/0.669
Resting Diastolic Blood Pressure (mmHg)	77.3 ± 10.4	76.10 ± 10.9	76.1 ± 9.3	76.3 ± 6.4	<0.001/0.932	<0.001/0.954	0.009/0.622
Resting Heart Rate (beats/min)	66.3 ± 10.3	65.5 ± 8.7	66.7 ± 10.5	66.8 ± 9.3	0.038/0.302	0.002/0.826	0.014/0.538
Performance Indices							
Counter-movement Jump Height (cm)	40.8 ± 5.6	42.5 ± 5.2 *^#^	40.9 ± 4.8	41.3 ± 3.2 ^#^	0.146/0.037	0.005/0.714	0.136/0.045
20 m Sprint (s)	3.12 ± 0.19	3.15 ± 0.16	3.26 ± 0.28	3.21 ± 0.25	0.003/0.773	0.056/0.208	0.056/0.209
Agility T-Test (s)	10.55 ± 0.43	10.37 ± 0.32 *^#^	10.56 ± 0.32	10.65 ± 0.35 ^#^	0.274/0.003	0.061/0.189	0.144/0.039
RAST Peak Power (W)	358.6 ± 50.3	364.7 ± 52.2	336.2 ± 56.9	326.3 ± 54.5	0.004/0.734	0.054/0.217	0.004/0.733
RAST Minimum Power (W)	238.8 ± 49.4	272.0 ± 55.2 *^#^	237.1 ± 47.0	230.1 ± 67.1 ^#^	0.485/<0.001	0.013/0.541	0.290/0.041
RAST Average Power (W)	294.6 ± 45.2	348.5 ± 50.8 *^#^	288.0 ± 47.5	281.2 ± 63.2 ^#^	0.295/0.002	0.054/0.216	0.362/0.003
RAST Fatigue Index, FI (%)	32.0 ± 6.4	24.1 ± 5.6 *^#^	27.6 ± 8.9	26.3 ± 9.4 ^#^	0.376/<0.001	<0.001/0.975	0.164/0.026
Yo-Yo IRL1 VO_2max_ (mL/kg/min)	43.4 ± 3.0	44.9 ± 4.3	43.7 ± 3.8	43.9 ± 4.2	0.117/0.064	0.002/0.802	0.064/0.178
Blood Lactate Concentration (mmol/L)	15.2 ± 7.7	14.3 ± 5.4	16.4 ± 5.6	16.0 ± 4.5	0.027/0.388	0.013/0.556	<0.001/0.907

Data presented as mean ± SD. (*) denotes significant differences between pre to post values in each group; (^#^) denotes significant differences between groups in the marked time point (*p* < 0.05).

## Data Availability

The data presented in this study are available from the corresponding author on reasonable request.
